# Health conditions and referral patterns among people experiencing homelessness: a 10-Year longitudinal study of specialized primary care in Hamburg, Germany

**DOI:** 10.1186/s12939-026-03071-y

**Published:** 2026-10-12

**Authors:** Daniel Tajdar, Dagmar Lühmann, Valentina Paucke, Matthias Augustin, Rachel Sommer, Brigitte Stephan, Juliane Traxler, Johanna Sophie Bobardt-Hartshorn, Thomas Kloppe, Martin Scherer, Carolin van der Leeden, Ingmar Schäfer

**Affiliations:** 1 Department of General Practice and Primary Care, University Medical Center Hamburg-Eppendorf, Martinistraße 52, D-20246 Hamburg, Germany; 2 Institute for Health Services Research in Dermatology and Nursing (IVDP), University Medical Center Hamburg-Eppendorf, Martinistraße 52, D-20246 Hamburg, Germany

**Keywords:** Homeless centers, Hospital referral, Diagnoses

## Abstract

**Background:**

Homelessness has increased substantially across Europe in recent years and is associated with a high burden of disease and premature mortality. However, longitudinal evidence on the spectrum of health conditions and healthcare referral patterns among people experiencing homelessness remains limited. This study aimed to describe the most common diagnoses in this population and examine their associations with referral to specialist outpatient care and referral to hospital.

**Methods:**

We conducted a retrospective longitudinal study using routinely collected clinical data from three specialized primary care centers for people experiencing homelessness in Hamburg, Germany. Data from 5,055 patients attending 13,330 consultations between June 5, 2013, and March 28, 2024, were analyzed. Diagnoses were coded by healthcare professionals according to ICD-10 criteria. The primary outcomes were referral to specialist outpatient care and referral to hospital. Associations between diagnoses and referral outcomes were assessed using regression analyses and are presented as odds ratios (ORs) with 95% confidence intervals (CIs).

**Results:**

The mean age of patients was 44.6 years (SD, 13.2), and 4,207 (83.2%) were male. Mental and behavioral disorders were the most frequently recorded diagnoses (1,436 patients, 28.4%), particularly disorders related to psychoactive substance use (817, 16.2%) and alcohol use (592, 11.7%). Overall, 10.9% of consultations resulted in referral, including 5.5% to specialist outpatient care and 5.4% with referral to hospital. Alcohol-related disorders (OR, 1.76; 95% CI, 1.39–2.23) and injuries and poisoning, including drug intoxication (OR, 1.64; 95% CI, 1.31–2.05), were significantly associated with referral to hospital. Referral to specialist outpatient care was more likely among older patients (OR per 10-year increase, 1.34; 95% CI, 1.22–1.47) and those presenting with musculoskeletal conditions. Most consultations (89.1%) did not result in a documented referral.

**Conclusions:**

Specialized primary care services were able to address the majority of health problems among people experiencing homelessness, with referral required in only a minority of consultations. Strengthening and integrating such services may improve access to care and support continuity of treatment across healthcare settings. Further research should evaluate interventions targeting mental health and substance use disorders, which represented the most common health concerns. Alcohol-related disorders were also associated with increased referral to hospital.

**Trial registration:**

Not applicable.

**Supplementary Information:**

The online version contains supplementary material available at https://doi.org/10.1186/s12939-026-03071-y.

## Background

Homelessness is a major public health challenge across Europe [1]. According to the European Typology of Homelessness and Housing Exclusion (ETHOS), homelessness includes people sleeping rough as well as those living in temporary or insecure accommodation [2]. Recent estimates suggest that more than one million people experienced homelessness in Germany in 2024, including approximately 56,000 people sleeping rough [3]. Although Germany is a high-income country with universal health coverage, people experiencing homelessness continue to face substantial barriers to accessing regular healthcare [4].

Homelessness is closely associated with poor health outcomes [5, 6]. Prolonged exposure to adverse living conditions, limited hygiene, inadequate nutrition, substance use, and injuries contributes to a high burden of acute and chronic disease among people experiencing homelessness [7–9]. Access to regular healthcare is often hindered by structural and individual barriers [10], including lack of health insurance, physical or mental incapacity, stigma, fear, and financial concerns [4, 8, 11, 12]. Consequently, healthcare is frequently delayed or underused, resulting in more advanced disease at presentation and poorer health outcomes [7, 13]. Overall, people experiencing homelessness have markedly poorer health than the general population [14], are three times more likely to have chronic diseases [15], and experience substantially higher mortality rates [8, 10, 16–19] and an increased risk of premature death [10, 20].

People experiencing homelessness are frequently stigmatized [5, 21] and report being treated disrespectfully by general practitioners [22, 23]. Many therefore seek medical care only in acute situations [21] and typically present to hospital emergency departments, where treatment cannot be refused [10, 24, 25]. This pattern not only increases pressure on emergency departments but also generates avoidable healthcare costs [6, 8, 26]. To address these challenges and improve access to care, several federal states and municipalities in Germany have established specialized medical centers in cooperation with non-governmental organizations [4, 27, 28]. In Hamburg, three such medical centers were established in 2013 [4]. These centers provide care by general practitioners and psychiatrists supported by medical assistants, nurses, and social workers [4]. Hamburg represents a typical urban European setting with a well-developed healthcare infrastructure, supporting the relevance of the findings for other European healthcare systems. Over the past decade, the characteristics and healthcare needs of people experiencing homelessness have evolved alongside demographic changes, healthcare policies, and service provision [1, 6]. These developments highlight the importance of longitudinal studies to better understand healthcare needs over extended periods rather than at a single point in time [6, 7].

Despite these developments, homeless patients frequently present with complex comorbidities and typically seek treatment only at advanced stages of illness [29], making a comprehensive understanding of their health problems essential. Although health problems among people experiencing homelessness have been widely studied, the available evidence remains fragmented. Many studies focus on specific conditions, particularly mental illness [30–34] or infectious diseases [35], thereby overlooking the broader spectrum of morbidity. Furthermore, much of the available literature is limited by methodological shortcomings, including small sample sizes (41 to 183 participants [32, 36–41]), often cross-sectional study designs [31, 40–43], or short observation periods [4, 27, 38, 44]. In many studies, diagnoses were based on self-reported information rather than clinical assessment [31, 32, 36, 42, 45, 46]. Consequently, high-quality longitudinal studies based on large patient populations investigating the association between clinically diagnosed illnesses and referral to specialist outpatient care or referral to hospital remain scarce [27].

To address these evidence gaps, this study aimed (1) to describe the most common diagnoses among patients receiving care at specialized medical centers for people experiencing homelessness and (2) to identify diagnoses recorded during consultations that were associated with referral to specialist outpatient care or referral to hospital. We therefore conducted a longitudinal analysis of routinely collected data from 5,055 patients comprising 13,330 consultations over a ten-year period in Hamburg.

## Methods

### Study design

We conducted a retrospective longitudinal study using routinely collected patient data from specialized primary care centers for homeless individuals in Hamburg, Germany. The study was approved by the Ethics Committee of the Hamburg Medical Association (file number PV4454) and supported by the Hamburg Social Welfare Authority (funding code 32771). It was performed in accordance with the Declaration of Helsinki (1964) and its subsequent amendments. Under German law (§ 75 SGB X), the evaluation of anonymized routine data is permissible without explicit patient consent. All patient records were de-identified during data extraction. The study covers the period from June 5, 2013, to March 28, 2024. This study was reported in accordance with the STROBE [47] reporting guideline.

### Setting

Data were obtained from three specialized medical centers that provided low-threshold primary medical and psychiatric care for people without permanent housing. All individuals seeking medical care were eligible for treatment regardless of health insurance status. Services included acute medical care and, where appropriate, support for integration into the regular healthcare system. All three centers were part of the same publicly funded project and followed a common care concept aimed at improving access to healthcare for people experiencing homelessness. While the centers differed in location, consultation hours, and the availability of psychiatric services, they operated within the same organizational framework. Across the three centers, care was delivered by multidisciplinary teams that included general practitioners, psychiatrists, nurses, medical assistants, and social workers. The participating general practitioners and psychiatrists had additional qualifications in addiction medicine, psychosomatic medicine, neurology, and palliative care. Doctors and medical staff were compensated on an hourly basis, whereas social workers were salaried without additional remuneration.

The first center was located at the Pik As overnight shelter in Hamburg-Neustadt, a central inner-city district. Weekly services included six hours of general medical consultations and six hours of psychiatric consultations. The care team consisted of two general practitioners, a psychiatrist, a specialist in psychiatry and psychotherapy, and two registered nurses.

The second center was set up at the Achterdwars men’s shelter in Hamburg-Bergedorf. It initially offered two weekly general medical sessions of three hours each, later reduced to one session per week, without psychiatric services. The staff included two general practitioners and a registered nurse.

The third center was located on Johanniswall in Hamburg’s old town. It offered two weekly general medical sessions, one lasting three hours and one two hours. A psychiatric consultation was offered every two weeks but was discontinued after six months due to low demand. Medical services were provided by two general practitioners, a medical assistant, and an experienced nurse.

### Participants

All patients attending one of the three centers between June 5, 2013, and March 28, 2024, were eligible for inclusion. Patients were excluded if age or sex information was missing or if recorded values were clearly implausible, for example because the recorded year of birth was not plausible. Consultations without a documented diagnosis were also excluded. The final sample comprised 5,055 patients with 13,330 consultations.

### Variables

The two primary outcomes were (1) referral to specialist outpatient care and (2) referral to hospital. Referral to specialist outpatient care was defined as a referral issued or initiated by the treating physician for further outpatient specialist care. Referral to hospital was defined as a referral to hospital issued or initiated by the treating physician. The available data did not allow further differentiation by specialist discipline, hospital type, or between planned hospital referrals and emergency transfers.

Independent variables included age, sex, and the diagnoses prompting consultations. Age was measured in years, with odds ratios calculated per 10-year increment, and sex was recorded as female or male. Diagnoses were coded by medical professionals using ICD-10 criteria. More than one diagnosis could be recorded for an individual patient.

### Data sources and measurement

All centers documented consultations electronically using x.vianova software (Medatixx, Germany). The records contained patient demographics, ICD-10 coded diagnoses, and information on referral to specialist outpatient care and referral to hospital.

### Statistical analysis

We conducted longitudinal analyses covering all consultations over more than ten years. Diagnoses were included if they met a minimum prevalence of 5% at the ICD-10 chapter, block, or category level [48]. To avoid overlap between nested ICD-10 categories, subcategories included separately in the regression models were excluded from their respective higher-level categories.

Sociodemographic data, healthcare use, and diagnoses were described on the patient level as means and standard deviations and as percentages. Associations between diagnoses and referral to specialist outpatient care and referral to hospital were analyzed on the consultation level using multivariable logistic regression with random effects at the patient level.

For an exploratory analysis of temporal differences, the observation period was divided into 2013–2018 and 2019–2024, and referral proportions between the two periods were compared using chi-square tests. We defined an α-level of 5% (*p* < .05) as statistically significant. All statistical analyses were performed using Stata version 15.1.

## Results

### Participants

**Fig. 1:**
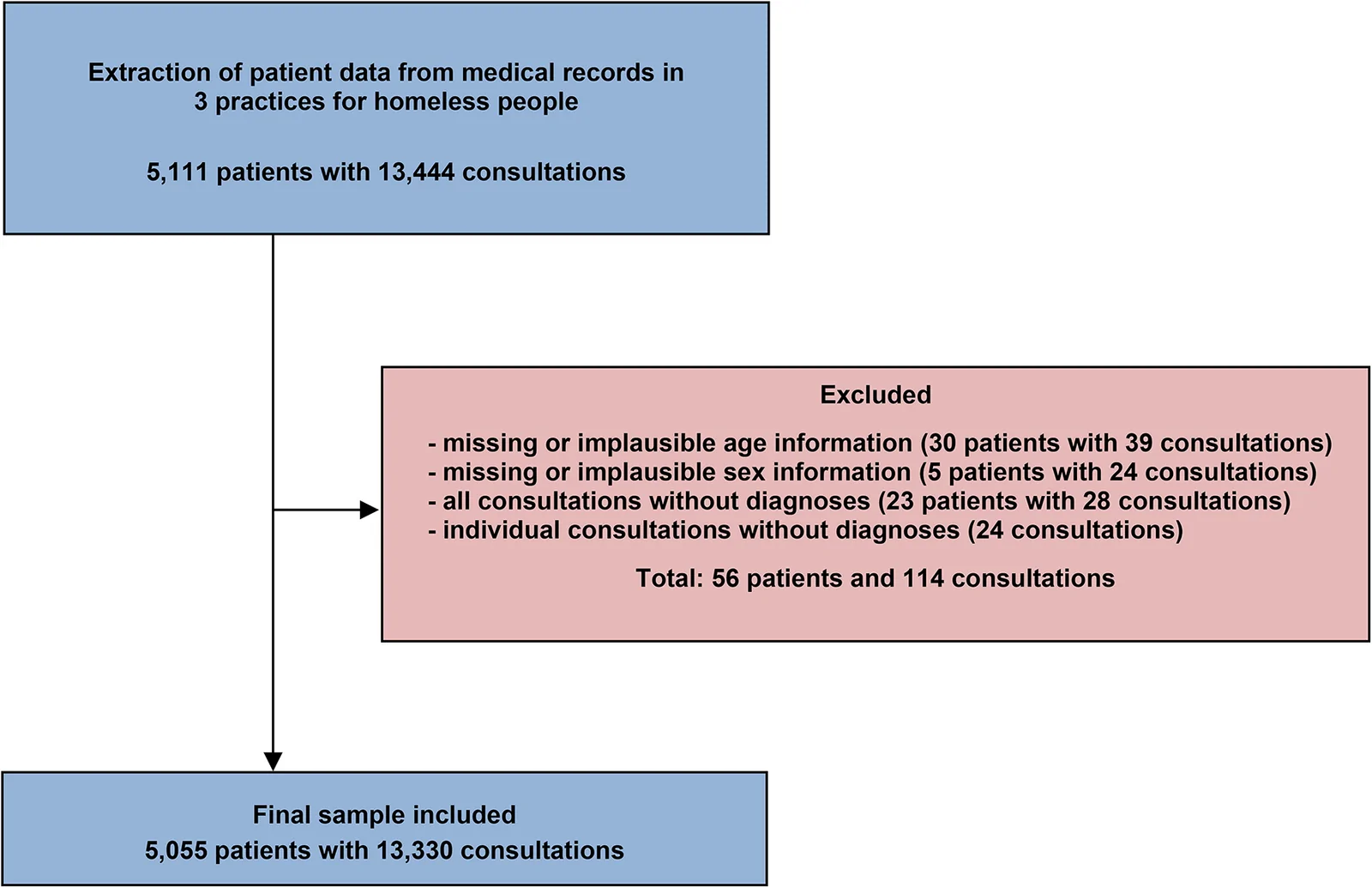
Flow Diagram of Study Sample Selection. Flow diagram illustrating the selection of the study population from the initial dataset. A total of 5,111 patients with 13,444 consultations were assessed for eligibility. Patients with missing or implausible age or sex information and consultations without documented diagnoses were excluded, resulting in a final sample of 5,055 patients with 13,330 consultations

The raw dataset comprised 5,111 patients with 13,444 consultations (Fig. 1). A total of 56 patients and 114 consultations were excluded. These exclusions included 30 patients with 39 consultations due to missing or implausible age information, five patients with 24 consultations due to missing or implausible sex information, and 23 patients with 28 consultations in which no diagnosis was documented. In addition, 24 individual consultations without a documented diagnosis were excluded. The final study population consisted of 5,055 patients with 13,330 consultations.

### Descriptive data

**Fig. 2:**
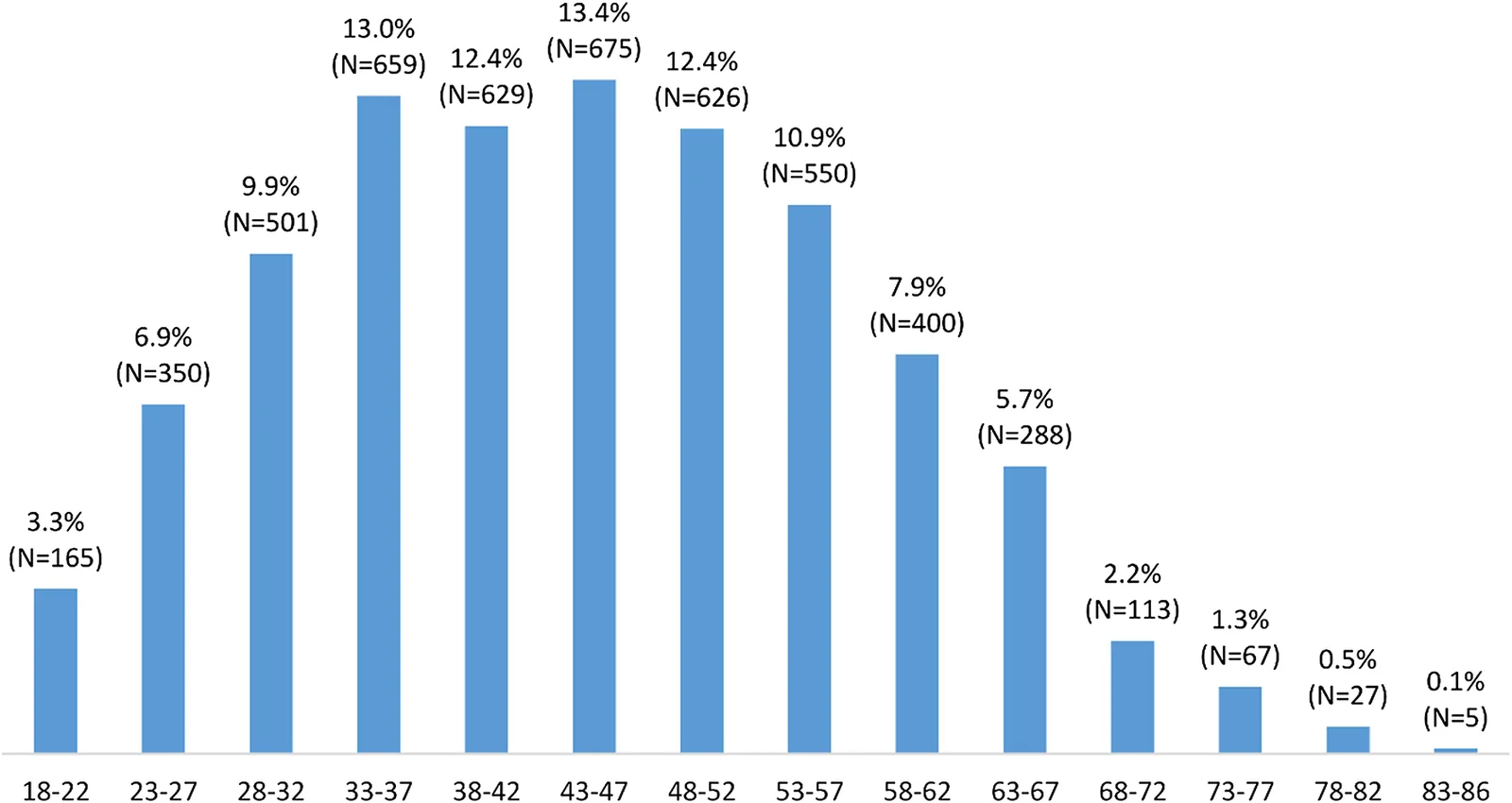
Age Distribution of the Study Population (in years). Distribution of 5,055 patients treated in specialized primary care centers for people experiencing homelessness. The figure shows the proportion of patients in each age group

Table 1 summarizes the sociodemographic characteristics and healthcare utilization of the included patients. The mean age was 44.6 years, and 4,207 patients (83.2%) were male. The age distribution is shown in Fig. 2. Most patients were between 28 and 57 years of age, with the largest age group being 43–47 years (13.4%; *n* = 675). Overall, 2,903 patients (57.4%) had only one consultation, whereas 2,152 patients (42.6%) had between two and 82 consultations during the observation period.

**Table 1:** Sociodemographic data and healthcare use

Characteristic	Sample (*n* = 5,055)
**Age** (in years):	
mean ± standard deviation	44.6 ± 13.2
**Sex**	
- male	4,207 (83.2%)
- female	848 (16.8%)
**Number of consultations**	
- 1	2,903 (57.4%)
- 2	857 (17.0%)
- 3-4	649 (12.8%)
- 5-6	248 (4.9%)
- 7-9	170 (3.4%)
- 10-12	89 (1.8%)
- 13-16	66 (1.3%)
- 17-20	28 (0.6%)
- 21-30	23 (0.5%)
- 31–82	22 (0.4%)
**Number of referrals to specialist outpatient care**	
- none	4,606 (91.1%)
- 1	315 (6.2%)
- 2	65 (1.3%)
- 3	44 (0.9%)
- 4-6	16 (0.3%)
- 7-10	9 (0.2%)
**Number of referrals to hospital**	
- none	4,454 (88.1%)
- 1	516 (10.2%)
- 2	65 (1.3%)
- 3	14 (0.3%)
- 4-5	4 (0.08%)
- 6-8	2 (0.04%)

**Fig. 3:**
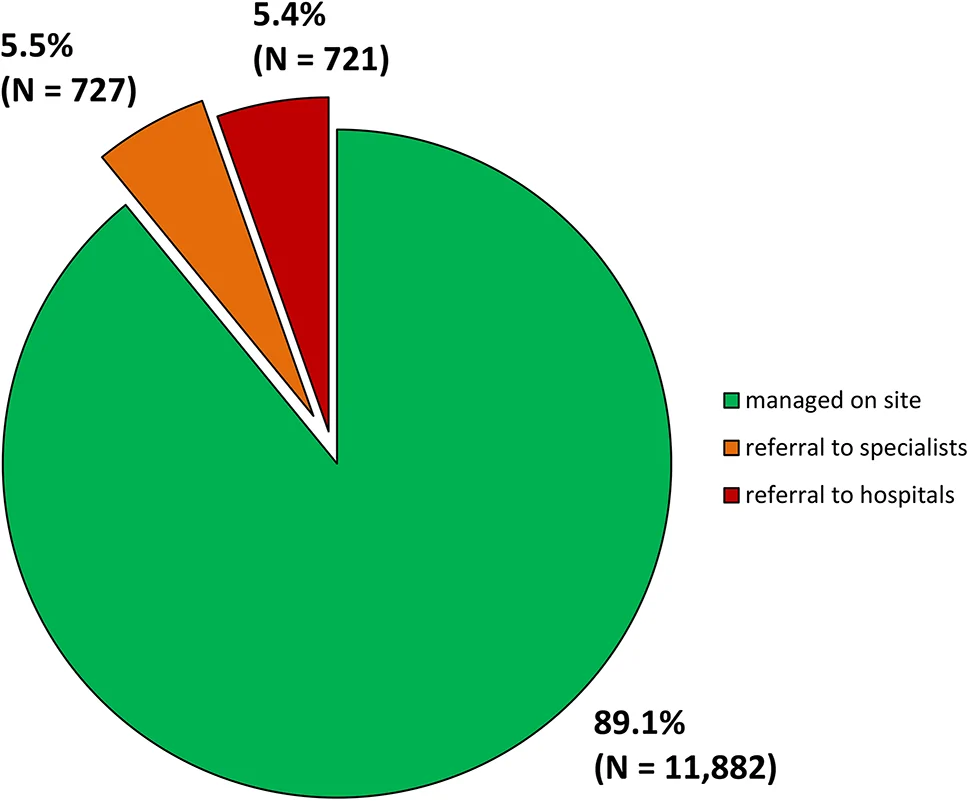
Healthcare Utilization and Referrals Among Homeless Patients. Distribution of 13,330 consultations among 5,055 patients treated in specialized primary care centers for people experiencing homelessness. The figure shows the proportion of consultations managed within primary care and those resulting in referral to specialist outpatient care or referral to hospital

A total of 727 referrals to specialist outpatient care (5.5% of consultations) were made for 449 patients (8.9% of the sample), and 721 referrals to hospital (5.4% of consultations) were made for 601 patients (11.9% of the sample) (Fig. 3).

Mental and behavioral disorders were the most prevalent diagnostic group (Table 2), affecting 1,436 patients (28.4%), including disorders due to psychoactive substance use (817 patients; 16.2%) and alcohol use (592 patients; 11.7%). A total of 184 patients (3.6%) had both a substance use disorder and another mental disorder documented during the observation period.

**Table 2:** Diagnoses*

ICD10-Code	Sample (*n* = 5,055)
**Mental and behavioral disorders (F00-F99)**	**1,436 (28.4%)**
- Mental and behavioral disorders due to psychoactive substance use (F10-F19)	817 (16.2%)
--- Mental and behavioral disorders due to use of alcohol (F10)	592 (11.7%)
- Neurotic, stress-related and somatoform disorders (F40-F48)	264 (5.2%)
**Diseases of the respiratory system (J00-J99)**	**1,187 (23.5%)**
- Acute upper respiratory infections (J00-J06)	586 (11.6%)
--- Acute upper respiratory infections of multiple andunspecified sites (J06)	501 (9.9%)
- Chronic lower respiratory diseases (J40-J47)	383 (7.6%)
**Certain infectious and parasitic diseases (A00-B99)**	**1**,**148 (22.7%)**
*- Pediculosis*,* acariasis and other infestation (B85-B89)*	*358 (7.1%)*
--- Pediculosis due to Pediculus humanus capitis (B85.0)	263 (5.2%)
*- Mycoses (B35-B49)*	*267 (5.3%)*
**Injury**,** poisoning and certain other consequences of external causes (S00-T98)**	**1**,**115 (22.1%)**
*- Injuries to unspecified part of trunk*,* limb or body region (T08-T14)*	*275 (5.4%)*
**Symptoms**,** signs and abnormal clinical and laboratory findings**,** not elsewhere classified (R00-R99)**	**993 (19.6%)**
*- Symptoms and signs involving the digestive system and abdomen (R10-R19)*	*351 (6.9%)*
--- Abdominal and pelvic pain (R10)	291 (5.8%)
*- Symptoms and signs involving the circulatory and respiratory systems (R00-R09)*	263 (5.2%)
*- General symptoms and signs (R50-R69)*	258 (5.1%)
**Diseases of the circulatory system (I00-I99)**	**923 (18.3%)**
*- Hypertensive diseases (I10-I15)*	*509 (10.1%)*
--- Essential (primary) hypertension (I10)	484 (9.6%)
**Diseases of the musculoskeletal system and connective tissue (M00-M99)**	**870 (17.2%)**
*- Dorsopathies (M40-M54)*	*420 (8.3%)*
--- Dorsalgia (M54)	348 (6.9%)
*- Arthropathies (M00-M25)*	*350 (6.9%)*
**Diseases of the digestive system (K00-K93)**	**742 (14.7%)**
*- Diseases of oesophagus*,* stomach and duodenum (K20-K31)*	*317 (6.3%)*
**Diseases of the skin and subcutaneous tissue (L00-L99)**	**677 (13.4%)**
**Endocrine**,** nutritional and metabolic diseases (E00-E89)**	**456 (9.0%)**
*- Diabetes mellitus (E10-E14)*	*298 (5.9%)*
--- Type 2 diabetes mellitus (E11)	256 (5.1%)
**Diseases of the nervous system (G00-G99)**	**394 (7.8%)**
*- Episodic and paroxysmal disorders (G40-G47)*	*299 (5.9%)*

*Diagnoses with prevalence < 5% are omitted

Detailed results on chronic diseases, comorbidities, consultations per patient, and diagnosis-specific referrals are provided in the supplementary tables (S1a–S1h). Patients with chronic conditions attended the centers more frequently. The mean number of consultations per patient was particularly high for chronic diseases of the circulatory system, including essential hypertension (3.97 ± 5.73 consultations per patient), chronic ischemic heart disease (3.18 ± 3.60), cerebrovascular diseases (4.11 ± 5.36), and diseases of arteries, arterioles, and capillaries (3.11 ± 2.46).

Similarly elevated consultation rates were observed for chronic respiratory diseases such as chronic obstructive pulmonary disease (2.96 ± 3.80) and asthma (2.85 ± 4.07), for chronic endocrine and metabolic diseases such as diabetes mellitus (4.14 ± 6.39), and for chronic skin diseases such as psoriasis (3.08 ± 3.73).

Overall, the study population was predominantly male, mental and behavioral disorders were the most prevalent diagnoses, and patients with chronic conditions tended to attend the centers more frequently.

### Main results

Associations between diagnoses and referrals are presented in Tables 3 and 4.

**Table 3:** Association between diagnoses and referrals to specialist outpatient care (*n* = 5,055; *N* = 13,330)

Characteristic	Odds ratio (95% CI)	*p*
**Age (per 10 years)**	**1.34 (1.22–1.47)**	**< 0.001**
Sex:		
- men	reference	
- women	1.10 (0.81***–***1.51)	0.540
Mental and behavioral disorders (F00-F99 excluding F10-F19 and F40-F48)	0.91 (0.66***–***1.27)	0.592
Mental and behavioral disorders due to psychoactive substance use (F10-F19 excluding F10)	1.34 (0.87***–***2.08)	0.185
Mental and behavioral disorders due to use of alcohol (F10)	0.78 (0.57***–***1.08)	0.131
Neurotic, stress-related and somatoform disorders (F40-F48)	0.96 (0.59***–***1.56)	0.877
Diseases of the respiratory system (J00-J99 excluding J00-J06 and J40-J47)	0.89 (0.57***–***1.39)	0.618
***Acute upper respiratory infections (J00-J06)***	***0.51 (0.33–0.79)***	***0.002***
Chronic lower respiratory diseases (J40-J47)	1.23 (0.88***–***1.73)	0.229
Certain infectious and parasitic diseases (A00-B99 excluding B85-B89 and B35-B49)	1.11 (0.81***–***1.51)	0.507
***Pediculosis***,*** acariasis and other infestation (B85-B89)***	***0.36 (0.20–0.65)***	***0.001***
Mycoses (B35-B49)	1.01 (0.62***–***1.64)	0.976
Injury, poisoning and certain other consequences of external causes (S00-T98 excluding T08-T14)	0.91 (0.68***–***1.21)	0.510
Injuries to unspecified part of trunk, limb or body region (T08-T14)	0.99 (0.56***–***1.76)	0.968
***Symptoms***,*** signs and abnormal clinical and laboratory findings***,*** not elsewhere classified (R00-R99 excluding R00-R19 and R50-R69)***	***2.67 (1.81–3.92)***	***< 0.001***
Symptoms and signs involving the digestive system and abdomen (R10-R19)	1.15 (0.76***–***1.74)	0.508
Symptoms and signs involving the circulatory and respiratory systems (R00-R09)	1.42 (0.92***–***2.18)	0.111
General symptoms and signs (R50-R69)	0.86 (0.55***–***1.35)	0.514
Diseases of the circulatory system (I00-I99 excluding I10-I15)	1.04 (0.77***–***1.39)	0.800
Hypertensive diseases (I10-I15)	0.76 (0.55***–***1.05)	0.093
***Dorsopathies (M40-M54)***	***1.42 (1.01–1.99)***	***0.045***
***Arthropathies (M00-M25)***	***2.43 (1.78–3.33)***	***< 0.001***
Diseases of the digestive system (K00-K93 excluding K20-K31)	1.36 (0.99***–***1.86)	0.056
Diseases of oesophagus, stomach and duodenum (K20-K31)	0.83 (0.54***–***1.26)	0.377
Diseases of the skin and subcutaneous tissue (L00-L99)	1.20 (0.89***–***1.63)	0.227
***Diabetes mellitus (E10-E14)***	***0.63 (0.40–0.97)***	***0.036***
Episodic and paroxysmal disorders (G40-G47)	1.11 (0.73***–***1.68)	0.629

95% CI: 95% confidence interval; p: probability value

**Table 4:** Association between diagnoses and referrals to hospital (*n* = 5,055; *N* = 13,330)

Characteristic	Odds ratio (95% CI)	*p*
Age (per 10 years)	1.04 (0.96***–***1.12)	0.368
Sex:		
- men	reference	
- women	1.04 (0.80***–***1.34)	0.782
Mental and behavioral disorders (F00-F99 excluding F10-F19 and F40-F48)	0.88 (0.66***–***1.18)	0.404
Mental and behavioral disorders due to psychoactive substance use (F10-F19 excluding F10)	1.32 (0.91***–***1.91)	0.138
***Mental and behavioral disorders due to use of alcohol (F10)***	***1.76 (1.39–2.23)***	***< 0.001***
Neurotic, stress-related and somatoform disorders (F40-F48)	0.72 (0.44***–***1.18)	0.189
Diseases of the respiratory system (J00-J99 excluding J00-J06 and J40-J47)	0.85 (0.58***–***1.25)	0.406
***Acute upper respiratory infections (J00-J06)***	***0.36 (0.23–0.57)***	***< 0.001***
***Chronic lower respiratory diseases (J40-J47)***	***0.56 (0.39–0.82)***	***0.003***
Certain infectious and parasitic diseases (A00-B99 excluding B85-B89 and B35-B49)	1.01 (0.76***–***1.34)	0.935
***Pediculosis***,*** acariasis and other infestation (B85-B89)***	***0.31 (0.18–0.54)***	***< 0.001***
***Mycoses (B35-B49)***	***0.42 (0.23–0.78)***	***0.006***
***Injury***,*** poisoning and certain other consequences of external causes *** ***(S00-T98 excluding T08-T14)***	***1.64 (1.31–2.05)***	***< 0.001***
Injuries to unspecified part of trunk, limb or body region (T08-T14)	0.70 (0.42***–***1.17)	0.172
***Symptoms***,*** signs and abnormal clinical and laboratory findings***,*** not elsewhere classified (R00-R99 excluding R00-R19 and R50-R69)***	***0.53 (0.31–0.89)***	***0.017***
***Symptoms and signs involving the digestive system and abdomen (R10-R19)***	***2.19 (1.60–3.01)***	***< 0.001***
***Symptoms and signs involving the circulatory and respiratory systems (R00-R09)***	***1.90 (1.30–2.79)***	***0.001***
General symptoms and signs (R50-R69)	1.39 (0.93***–***2.07)	0.106
***Diseases of the circulatory system (I00-I99 excluding I10-I15)***	***2.40 (1.90–3.05)***	***< 0.001***
***Hypertensive diseases (I10-I15)***	***0.50 (0.36–0.68)***	***< 0.001***
***Dorsopathies (M40-M54)***	***0.43 (0.27–0.67)***	***< 0.001***
***Arthropathies (M00-M25)***	***0.36 (0.22–0.58)***	***< 0.001***
***Diseases of the digestive system (K00-K93 excluding K20-K31)***	***1.43 (1.08–1.89)***	***0.012***
Diseases of oesophagus, stomach and duodenum (K20-K31)	0.66 (0.43***-***1.00)	0.050
***Diseases of the skin and subcutaneous tissue (L00-L99)***	***0.58 (0.43–0.80)***	***0.001***
Diabetes mellitus (E10-E14)	0.70 (0.48***–***1.02)	0.061
Episodic and paroxysmal disorders (G40-G47)	0.80 (0.54***–***1.18)	0.259

95% CI: 95% confidence interval; p: probability value

Overall, referral to specialist outpatient care was mainly associated with older age, musculoskeletal disorders, and nonspecific clinical and laboratory findings, whereas referral to hospital was more frequent in patients with alcohol-related disorders, injuries and poisoning, and several digestive and circulatory conditions.

A higher probability of referral to specialist outpatient care was associated with increasing age (odds ratio per 10-year difference, 1.34; 95% confidence interval, 1.22–1.47; *P* < .001) and with musculoskeletal disorders, specifically arthropathies (2.43; 1.78–3.33; *P* < .001) and dorsopathies (1.42; 1.01–1.99; *P* = .045). Referrals were also more frequent in patients with nonspecific clinical and laboratory findings (2.67; 1.81–3.92; *P* < .001).

A higher likelihood of referral to hospital was observed for mental and behavioral disorders due to alcohol use (1.76; 1.39–2.23; *P* < .001) and for injury, poisoning, and certain other consequences of external causes (1.64; 1.31–2.05; *P* < .001). Increased referral rates were also seen for digestive system conditions, including diseases of the digestive system (1.43; 1.08–1.89; *P* = .012) and symptoms and signs involving the digestive system and abdomen (2.19; 1.60–3.01; *P* < .001). Furthermore, referral to hospital was more likely in patients with diseases of the circulatory system (2.40; 1.90–3.05; *P* < .001) and with symptoms and signs involving the circulatory and respiratory systems (1.90; 1.30–2.79; *P* = .001).

Conversely, several diagnoses were associated with lower referral rates. Both specialist outpatient and hospital referrals were less likely in patients with acute upper respiratory infections (0.51; 0.33–0.79; *P* = .002 and 0.36; 0.23–0.57; *P* < .001, respectively) and pediculosis, acariasis, and other infestations (0.36; 0.20–0.65; *P* = .001 and 0.31; 0.18–0.54; *P* < .001).

A lower probability of referral to specialist outpatient care was also observed for diabetes mellitus (0.63; 0.40–0.97; *P* = .036). Lower probabilities of referral to hospital were found for hypertensive diseases (0.50; 0.36–0.68; *P* < .001), chronic lower respiratory diseases (0.56; 0.39–0.82; *P* = .003), mycoses (0.42; 0.23–0.78; *P* = .006), and diseases of the skin and subcutaneous tissue (0.58; 0.43–0.80; *P* = .001).

Overall, referral patterns differed considerably between diagnostic groups, with some conditions being more frequently managed without referral, whereas others more often resulted in referral to specialist outpatient care or hospital.

To examine potential changes over time, we divided the observation period into two consecutive periods: 2013–2018 (4,806 consultations) and 2019–2024 (8,524 consultations). The proportion of consultations resulting in referral to specialist outpatient care was 5.6% (*n* = 271) in the first period and 5.4% (*n* = 456) in the second period, with no statistically significant difference (*p* = .480). In contrast, the proportion of consultations resulting in referral to hospital increased significantly from 4.9% (*n* = 233) in the first period to 5.7% (*n* = 488) in the second period (*p* = .032).

## Discussion

### Statement of principal findings

Our study shows that mental health problems and addiction were the most frequent conditions among people experiencing homelessness. Nearly one in three patients had a recorded mental and behavioral disorder, many of them linked to alcohol or other psychoactive substances. Alcohol-related disorders were also associated with a higher likelihood of referral to hospital.

Despite this burden, the majority of health problems were managed within specialized, low-threshold primary care services. Only 10.9% of consultations resulted in a documented referral, suggesting that many health problems could be managed within the specialized primary care centers.

Referral patterns differed by diagnosis. Musculoskeletal conditions and nonspecific clinical and laboratory findings were associated with a higher likelihood of referral to specialist outpatient care, suggesting that these conditions more often required diagnostic assessment or treatment beyond the services available at the centers. In contrast, the lower referral rates observed for several common conditions, such as acute upper respiratory infections and infestations, may indicate that these conditions could often be managed within the centers. However, the reasons for individual referral decisions were not available in our data. Patients with chronic conditions also attended the centers more frequently, highlighting the need for ongoing management of chronic diseases.

### Strengths and limitations

Homeless individuals belong to so-called “hard-to-reach” populations, alongside people with mental illness, sex workers, and people who use drugs [49]. Consequently, earlier studies on the prevalence of disease among homeless people often relied on relatively small samples, typically ranging from 41 to 183 participants [32, 36–41], and even the larger investigations included only 232 to 651 individuals [31, 42–44, 46, 50]. With 5,055 participants, our study is among the largest in this field [27, 51].

With an observation period of more than ten years, our study was also among those with the longest duration [50, 51], whereas most previous studies used a cross-sectional design [31, 40–43], or shorter observation periods of several months to two years [4, 27, 38, 44]. Long-term analyses provide a more reliable picture of disease prevalence and better capture chronic conditions and comorbidities. For example, an earlier analysis of a subset of this population included 840 individuals and focused only on first consultations in 2013 and 2014 [4]. In contrast, 42.6% of patients in our study had repeated contacts, with up to 82 consultations.

In the present study, acute and chronic diseases and comorbidities were coded by medical professionals, whereas many earlier studies relied on patient-reported diagnoses [31, 32, 36, 42, 45, 46].

As systematic reviews have shown, many previous investigations focused on specific conditions such as mental illness [30, 34] or infectious diseases [35]. In contrast, our study used a comprehensive dataset covering more than a decade of primary care, reflecting real-world conditions and allowing a broad assessment of health problems. We analyzed data using multivariable regression, accounting for age, sex, and repeated consultations within patients.

However, the retrospective use of routine clinical data has several limitations. Documentation practices may have varied between clinicians and centers and over time, and the reported diagnostic data might therefore be incomplete or imprecise because they could not be clinically verified. The available data did not allow us to account for illness severity or individual social circumstances that may have influenced healthcare use and referral decisions. Psychiatric diagnoses were also coded by general practitioners and nurses based on ICD-10 criteria, which may have led to underestimation of severe mental illnesses and may not fully capture the complexity of psychiatric morbidity. The routine data did not allow a separate analysis of psychiatric service attendance, psychiatric referral pathways, or the characteristics of patients attending psychiatric consultations. It was not possible to compare the homeless population to a general practice population.

Although we explored referral rates across two broad time periods, we did not examine specific secular changes in healthcare services, policies, homelessness patterns, substance use trends, or broader social and public health conditions that may have influenced referral practices over the ten-year observation period.

Information on how patients accessed the specialized medical centers (e.g., self-referral or outreach) and whether patient populations overlapped across centers was not routinely documented and therefore could not be evaluated. In addition, the available routine data did not allow clinic-specific patient counts or referral analyses. We could therefore not report the proportion of patients attending each center or assess whether referral rates differed between centers. At some centers, however, social workers supported access by reminding patients of appointments, motivating them to attend, or accompanying them to consultations.

Nevertheless, we could not determine whether patients selected the specialized centers based on the services available or sought care elsewhere for conditions requiring other specialties. Such self-selection may have influenced the observed referral rates.

Housing status was not available longitudinally, and we could therefore not determine whether patients remained homeless throughout the observation period or whether changes in housing status affected their continued use of the specialized centers. This may have influenced the observed number of consultations per patient.

In addition, our study included only individuals who attended the specialized medical centers and may therefore not be representative of the broader population experiencing homelessness. People who did not access these services, including those with unmet mental health needs, are not represented in the dataset. This may limit the generalizability of our findings and may have introduced selection bias.

We could not assess whether excluded patients or consultations differed systematically from those included in the analysis. Although the number of excluded records was small, the exclusion of records with missing or implausible sociodemographic information or without documented diagnoses may have introduced selection bias.

We were also unable to determine whether referrals were appropriate, whether referred individuals subsequently attended specialist outpatient care or hospital services, or what care they received following referral. The available data did not allow us to distinguish between different specialist disciplines or types of hospital services, or between planned hospital referrals and emergency transfers. The referral rate may be underestimated, as some patients may have accessed emergency care without formal referral or full documentation, and we could not distinguish between clinician decisions and missed referrals due to patient refusal.

### Comparison with the literature

High-quality studies with large samples and extended observation periods that cover the full spectrum of health problems in homeless populations, and their association with referral to specialists and referral to hospital, remain rare.

One of the few comparable investigations is the study by Kunstmann et al., which reported on the medical treatment of 5,276 homeless people at five locations in North Rhine-Westphalia over 16 months [27]. According to the authors, 9.4% of all patient contacts resulted in referral to specialist outpatient care [27]. In our analysis, 5.5% of all patient contacts were referred to specialist outpatient care, with referrals associated with older age, musculoskeletal problems, and nonspecific clinical and laboratory findings. The higher referral rate for nonspecific clinical and laboratory findings may reflect the need for further diagnostic assessment when a specific diagnosis could not be established within the centers.

Kunstmann et al. further reported that 8% of all patients were referred to hospitals, most frequently for detoxification programs [27]. In our study, 11.9% of patients had at least one referral to hospital, corresponding to 5.4% of all consultations. Referral to hospital was associated with alcohol-related disorders, injuries and poisoning (including drug intoxication), as well as diseases of the digestive and circulatory systems and symptoms involving the circulatory and respiratory systems. These associations may reflect a greater need for hospital-based assessment or treatment in these diagnostic groups. Conversely, several common conditions, including acute upper respiratory infections, infestations, and some chronic conditions, were associated with lower referral rates. This may indicate that these conditions could often be managed within the specialized primary care centers without referral. However, lower referral rates should not necessarily be interpreted as reflecting less severe disease, as referral decisions may also have been influenced by individual clinical circumstances, patient preferences, or barriers to accessing further care.

Overall, 10.9% of patient contacts resulted in referral. Consistent with previous findings, this suggests that most consultations can be managed within specialized medical centers without referral [27], highlighting their role in providing effective care and reducing unnecessary hospital use [52, 53].

Individuals experiencing homelessness are disproportionately affected by chronic diseases [15] and show higher rates of multimorbidity than the general population [54, 55]. Barriers to care often lead to late presentation, when chronic conditions have already resulted in complications [29]. Consistent with this, patients with chronic conditions in our study attended the medical centers more frequently. For example, the mean number of consultations per patient was twice as high among those with diabetes mellitus (4.14 ± 6.39) than among patients with infectious diseases (1.93 ± 1.91), underscoring the need for effective chronic disease management.

The systematic review by Gutwinski et al. reported high rates of mental disorders, particularly alcohol and drug use disorders [34]. Consistent with these findings, mental and behavioral disorders were the most frequent diagnostic group in our study (28.4% of patients), including disorders due to psychoactive substance use (16.2%) and alcohol use (11.7%). Alcohol-related disorders were also associated with a higher likelihood of referral to hospital.

This pattern is supported by evidence from the United Kingdom, where emergency department visits among homeless people were associated with substance and alcohol dependence [54], as well as findings from the United States showing higher hospitalization rates for these conditions compared with non-homeless populations [56]. In Hamburg, drug intoxication is the leading cause of death among homeless individuals [57].

The high prevalence of mental and behavioral disorders is particularly relevant in view of the different use of psychiatric services across the centers. While psychiatric consultations at the Johanniswall center were discontinued after six months because of low attendance, this should not necessarily be interpreted as indicating a low need for psychiatric care. At Pik As, psychiatric care relied strongly on outreach and close collaboration between psychiatrists and social workers. Patients could be approached in their rooms, attend consultations independently, or be accompanied by social workers. Care often focused on building trust, establishing contact, and motivating patients to engage in treatment, particularly in the presence of barriers such as lack of insight into illness, fear or mistrust of psychiatric care, and severe chronic mental illness. The lower attendance at Johanniswall may therefore have been influenced by differences in service organization and integration with social services rather than by a low need for psychiatric care.

In addition to mental disorders, homeless people experience a range of somatic diseases related to living conditions [7]. Previous reviews reported infectious, cardiovascular, respiratory diseases, and injuries as common conditions [8]. In our study, respiratory diseases, infections, and injuries were among the most frequent somatic conditions.

Around 80% of the homeless population are men [30, 42], which is consistent with our findings (83.2%). The lower proportion of women in our study may partly reflect gender-specific patterns of homelessness and the use of support services. Homeless women are more likely to remain in less visible forms of homelessness and are more frequently reliant on informal accommodations with friends, relatives, or acquaintances, which can reduce their contact with conventional support services for the homeless [58, 59]. Barriers to low-threshold healthcare for women experiencing homelessness include mistrust and shame, limited availability of gender-specific services, and concerns about male-dominated care settings [59]. We could not determine whether women were less likely than men to use the specialized centers, while the location of the centers may also have contributed to the high proportion of male patients. Future studies should examine gender-specific differences in the use of support services and access to specialized health care.

The mean age in our study (44.6 years) is consistent with reports from other metropolitan areas in Europe [27, 31, 42, 44, 50].

### Implications for clinical practice

Mental and behavioral disorders were the most frequent diagnostic group, while alcohol-related disorders were associated with referral to hospital. The experience across the centers suggests that psychiatric care for people experiencing homelessness may require a flexible, low-threshold approach, including close collaboration between psychiatric and social care and, where appropriate, outreach services.

Primary care should emphasize systematic screening, early involvement of psychosocial services, and integration with addiction treatment.

The higher consultation frequency among patients with chronic conditions indicates the need for structured long-term management. Continuity of care and follow-up are essential.

Certain diagnostic groups, particularly musculoskeletal conditions, were more often referred to outpatient care, highlighting the need for clear referral pathways to ensure timely treatment and continuity of care.

Integrating specialized services into primary care and strengthening links with addiction and social care may improve continuity of care across healthcare settings.

## Conclusions

In this longitudinal study, most consultations did not result in a documented referral. Specialized services enabled access to care and provided referral pathways to specialist outpatient and hospital care. Expanding and integrating these services may improve access and continuity of care across healthcare settings. Future research should evaluate interventions targeting mental health and substance use, which were highly prevalent. Alcohol-related disorders were also strongly associated with referral to hospital.

## Supplementary Information

Below is the link to the electronic supplementary material.

Supplementary Material 1

## Data Availability

The datasets used and analyzed during the current study are available from the corresponding author on reasonable request.

## Abbreviations

CI: Confidence Interval
ICD: 10–International Statistical Classification of Diseases and Related Health Problems, 10th Revision
OR: Odds Ratio
SD: Standard Deviation
ETHOS: European Typology of Homelessness and Housing Exclusion
SGB X: Sozialgesetzbuch, Book X (German Social Code, Book X)
STROBE: Strengthening the Reporting of Observational Studies in Epidemiology
