# Supplementary material for: Health conditions and referral patterns among people experiencing homelessness: a 10-Year longitudinal study of specialized primary care in Hamburg, Germany
Source: Int J Equity Health. 2026 Oct 12;25:268. doi: 10.1186/s12939-026-03071-y (PMC13732960; doi:10.1186/s12939-026-03071-y)
Supplement: Supplementary file 1 [file 12939_2026_3071_MOESM1_ESM.docx]

**Table S1a: Number of patients, consultations, comorbid diagnoses, referrals to outpatient care and hospital admissions by ICD10 classification**

| **ICD10 codes** | **Number of patients** | **Number of consultations** | **Consultations per patient** | **Comorbid diagnoses** | **Outpatient referrals** | **Hospital admissions** |
| --- | --- | --- | --- | --- | --- | --- |
| ***Mental and behavioral disorders (F00-F99)*** | ***1,436 (28.4%)*** | ***3,530 (26.5%)*** | ***2.46 ± 3.09*** | ***3.23 ± 2.68*** | ***206 (5.8%)*** | ***238 (6.7%)*** |
| - … due to psychoactive substance use (F10-F19) | 817 (16.2%) | 2,151 (16.0%) | 2.63 ± 3.45 | 3.77 ± 2.85 | 124 (5.8%) | 176 (8.2%) |
| *--- … due to use of alcohol (F10)  --- … due to multiple drug use and use of other psychoactive substances (F19)  --- … due to use of tobacco (F17)* | *592 (11.7%) 176 (3.5%) 54 (1.1%)* | *1,716 (12.9%) 336 (2.5%) 123 (0.9%)* | *2.90 ± 3.89 1.91 ± 1.53 2.28 ± 1.77* | *3.87 ± 2.96 3.43 ± 1.88 6.93 ± 4.15* | *94 (5.5%) 16 (4.8%) 27 (22.0%)* | *144 (8.4%) 27 (8.0%) 9 (7.3%)* |
| - Mood disorders (F30-F39) | 271 (5.4%) | 623 (4.6%) | 2.27 ± 2.40 | 3.50 ± 3.41 | 43 (7.0%) | 39 (6.3%) |
| *--- Depressive episode (F32) --- Recurrent depressive disorder (F33)* | *195 (3.9%) 57 (1.1%)* | *424 (3.2%) 150 (1.1%)* | *2.17 ± 2.40 2.63 ± 2.35* | *3.38 ± 2.74 4.37 ± 5.14* | *27 (6.4%) 16 (10.7%)* | *23 (5.4%) 15 (10.0%)* |
| - Neurotic, stress-related and somatoform disorders (F40-F48) | 264 (5.2%) | 520 (3.9%) | 1.96 ± 2.08 | 3.19 ± 3.02 | 37 (7.2%) | 21 (4.1%) |
| *--- Somatoform disorders (F45)  --- Severe stress, and adjustment disorders (F43) --- Other anxiety disorders (F41)* | *102 (2.0%) 83 (1.6%) 71 (1.4%)* | *179 (1.3%) 129 (1.0%) 176 (1.3%)* | *1.75 ± 1.36 1.55 ± 1.41 2.48 ± 3.04* | *3.52 ± 3.77 2.78 ± 2.34 2.93 ± 1.59* | *17 (9.7%) 8 (6.2%) 7 (3.9%)* | *10 (5.7%) 6 (4.7%) 3 (1.7%)* |
| - Schizophrenia, schizotypal and delusional disorders (F20-F29) | 240 (4.8%) | 487 (3.6%) | 2.01 ± 1.91 | 2.35 ± 2.25 | 23 (4.8%) | 24 (5.0%) |
| *--- Schizophrenia (F20) --- Unspecified nonorganic psychosis (F29)* | *151 (3.0%) 58 (1.2%)* | *296 (2.2%) 110 (0.8%)* | *1.96 ± 1.88 1.90 ± 1.47* | *2.12 ± 2.10 2.48 ± 1.91* | *10 (3.4%) 3 (2.7%)* | *13 (4.4%) 6 (5.5%)* |
| - Disorders of adult personality and behavior (F60-F69) | 62 (1.2%) | 126 (1.0%) | 2.03 ± 1.39 | 3.30 ± 2.67 | 9 (7.1%) | 7 (5.6%) |
| ***Diseases of the respiratory system (J00-J99)*** | ***1,187 (23.5%)*** | ***2,571 (19.3%)*** | ***2.17 ± 2.74*** | ***2.97 ± 2.60*** | ***136 (5.3%)*** | ***89 (3.5%)*** |
| - Acute upper respiratory infections (J00-J06) | 586 (11.6%) | 962 (7.2%) | 1.64 ± 1.35 | 2.68 ± 2.62 | 30 (3.1%) | 22 (2.3%) |
| *--- … of multiple and unspecified sites (J06)* | *501 (9.9%)* | *794 (6.0%)* | *1.58 ± 1.27* | *2.54 ± 2.10* | *20 (2.5%)* | *18 (2.3%)* |
| - Chronic lower respiratory diseases (J40-J47) | 383 (7.6%) | 1,077 (8.1%) | 2.81 ± 3.69 | 3.61 ± 3.29 | 82 (7.6%) | 41 (3.8%) |
| *--- Other chronic obstructive pulmonary disease (J44) --- Bronchitis, not specified as acute or chronic (J40) --- Asthma (J45)* | *225 (4.5%) 103 (2.0%) 54 (1.1%)* | *666 (5.0%) 224 (1.7%) 154 (1.2%)* | *2.96 ± 3.80 2.17 ± 1.97 2.85 ± 4.07* | *3.67 ± 3.08 3.88 ± 4.66 2.95 ± 1.81* | *56 (8.4%) 18 (8.0%) 3 (2.0%)* | *29 (4.4%) 2 (0.9%) 10 (6.5%)* |

**Table S1b: Number of patients, consultations, comorbid diagnoses, referrals to outpatient care and hospital admissions by ICD10 classification (continued)**

| **ICD10 codes** | **Number of patients** | **Number of consultations** | **Consultations per patient** | **Comorbid diagnoses** | **Outpatient referrals** | **Hospital admissions** |
| --- | --- | --- | --- | --- | --- | --- |
| - Other acute lower respiratory infections (J20-J22) | 158 (3.1%) | 291 (2.2%) | 1.84 ± 1.51 | 3.12 ± 3.09 | 14 (4.8%) | 7 (2.4%) |
| *--- Acute bronchitis (J20)* | *156 (3.1%)* | *289 (2.2%)* | *1.85 ± 1.52* | *3.14 ± 3.09* | *14 (4.8%)* | *7 (2.4%)* |
| - Other diseases of upper respiratory tract (J30-J39) | 111 (2.2%) | 202 (1.5%) | 1.82 ± 1.45 | 3.04 ± 2.63 | 17 (8.4%) | 15 (7.4%) |
| *--- Vasomotor and allergic rhinitis (J30)* | *55 (1.1%)* | *106 (0.8%)* | *1.93 ± 1.60* | *3.08 ± 3.02* | *9 (8.5%)* | *2 (1.9%)* |
| - Other diseases of the respiratory system (J95-J99) | 79 (1.6%) | 124 (0.9%) | 1.57 ± 0.76 | 2.32 ± 1.26 | 2 (1.6%) | 1 (0.8%) |
| *--- Other respiratory disorders (J98)* | *76 (1.5%)* | *120 (0.9%)* | *1.58 ± 0.77* | *2.23 ± 1.14* | *2 (1.7%)* | *0 (-)* |
| ***Certain infectious and parasitic diseases (A00-B99)*** | ***1,148 (22.7%)*** | ***2,211 (16.6%)*** | ***1.93 ± 1.91*** | ***3.20 ± 2.94*** | ***124 (5.6%)*** | ***97 (4.4%)*** |
| - Pediculosis, acariasis and other infestations (B85-B89) | 358 (7.1%) | 658 (4.9%) | 1.84 ± 1.74 | 3.12 ± 3.18 | 17 (2.6%) | 16 (2.4%) |
| *--- Pediculosis and phthiriasis (B85) --- Scabies (B86)* | *263 (5.2%) 108 (2.1%)* | *467 (3.5%) 194 (1.5%)* | *1.78 ± 1.43 1.80 ± 1.60* | *2.76 ± 2.44 3.98 ± 4.37* | *11 (2.4%) 6 (3.1%)* | *7 (1.5%) 8 (4.1%)* |
| - Mycoses (B35-B49) | 267 (5.3%) | 475 (3.6%) | 1.78 ± 1.55 | 3.42 ± 3.33 | 32 (6.7%) | 12 (2.5%) |
| *---* *Unspecified mycosis (B49) --- Dermatophytosis (B35) --- Other superficial mycoses (B36)* | *111 (2.2%) 101 (2.0%) 58 (1.2%)* | *178 (1.3%) 194 (1.5%) 110 (0.8%)* | *1.60 ± 1.21 1.92 ± 1.59 1.90 ± 1.61* | *3.26 ± 2.71 3.74 ± 4.25 4.22 ± 4.94* | *8 (4.5%) 14 (7.2%) 15 (13.6%)* | *3 (1.7%) 3 (1.6%) 4 (3.6%)* |
| - Intestinal infectious diseases (A00-A09) | 141 (2.8%) | 308 (2.3%) | 2.18 ± 2.10 | 4.14 ± 3.80 | 15 (4.9%) | 12 (3.9%) |
| *--- Other gastroenteritis and colitis of infectious and unspecified origin (A09)* | *88 (1.7%)* | *177 (1.3%)* | *2.01 ± 2.08* | *3.15 ± 1.70* | *4 (2.3%)* | *5 (2.8%)* |
| - Viral infections characterized by skin and mucous membrane lesions (B00-B09) | 137 (2.7%) | 209 (1.6%) | 1.53 ± 1.18 | 2.93 ± 3.45 | 19 (9.1%) | 3 (1.4%) |
| *--- Other such infections, not elsewhere classified (B08)* | *52 (1.0%)* | *72 (0.5%)* | *1.38 ± 0.99* | *2.19 ± 1.18* | *1 (1.4%)* | *1 (1.4%)* |
| - Other bacterial diseases (A30-A49) | 103 (2.0%) | 183 (1.4%) | 1.78 ± 1.20 | 3.70 ± 3.07 | 9 (4.9%) | 24 (13.1%) |
| *--- Erysipelas (A46)* | *69 (1.4%)* | *121 (0.9%)* | *1.75 ± 1.18* | *3.89 ± 3.55* | *5 (4.1%)* | *18 (14.9%)* |
| - Other viral diseases (B25-B34) | 85 (1.7%) | 123 (0.9%) | 1.45 ± 0.79 | 2.01 ± 1.34 | 4 (3.3%) | 3 (2.4%) |

**Table S1c: Number of patients, consultations, comorbid diagnoses, referrals to outpatient care and hospital admissions by ICD10 classification (continued)**

| **ICD10 codes** | **Number of patients** | **Number of consultations** | **Consultations per patient** | **Comorbid diagnoses** | **Outpatient referrals** | **Hospital admissions** |
| --- | --- | --- | --- | --- | --- | --- |
| *--- Viral infection of unspecified site (B34)* | *80 (1.6%)* | *116 (0.9%)* | *1.45 ± 0.81* | *2.00 ± 1.37* | *2 (1.7%)* | *3 (2.6%)* |
| - Viral hepatitis (B15-B19) | 75 (1.5%) | 156 (1.2%) | 2.08 ± 1.71 | 3.99 ± 1.93 | 23 (14.7%) | 9 (5.8%) |
| *--- Chronic viral hepatitis (B18)* | *60 (1.2%)* | *137 (1.0%)* | *2.28 ± 1.83* | *4.14 ± 1.93* | *23 (16.8%)* | *8 (5.8%)* |
| - Tuberculosis (A15-A19) | 62 (1.2%) | 114 (0.9%) | 1.84 ± 1.26 | 4.00 ± 3.83 | 9 (7.9%) | 17 (14.9%) |
| ***Injury, poisoning and other consequences of external causes (S00-T98)*** | ***1,115 (22.1%)*** | ***2,157 (16.2%)*** | ***1.93 ± 2.22*** | ***2.92 ± 2.84*** | ***105 (4.9%)*** | ***157 (7.3%)*** |
| - Injuries to the head (S00-S09) | 186 (3.7%) | 296 (2.2%) | 1.59 ± 1.23 | 3.21 ± 2.28 | 10 (3.4%) | 23 (7.8%) |
| *--- Superficial injury of head (S00) --- Open wound of head (S01)* | *75(1.5%) 70 (1.4%)* | *104 (0.8%) 125 (0.9%)* | *1.39 ± 0.85 1.79 ± 1.21* | *2.85 ± 2.32 3.48 ± 2.48* | *5 (4.8%) 2 (1.6%)* | *8 (7.7%) 8 (6.4%)* |
| - Injuries to the thorax (S20-S29) | 103 (2.0%) | 160 (1.2%) | 1.55 ± 0.89 | 3.90 ± 4.22 | 8 (5.0%) | 22 (13.8%) |
| *--- Superficial injury of thorax (S20) --- Fracture of rib(s), sternum and thoracic spine (S22)* | *52 (1.0%) 52 (1.0%)* | *90 (0.7%) 74 (0.6%)* | *1.73 ± 1.12 1.42 ± 0.61* | *4.70 ± 5.24 2.96 ± 1.96* | *6 (6.7%) 3 (4.1%)* | *9 (10.0%) 15 (20.3%)* |
| - Injuries to the wrist and hand (S60-S69) | 192 (3.8%) | 320 (2.4%) | 1.67 ± 1.46 | 2.65 ± 1.67 | 18 (5.6%) | 39 (12.2%) |
| *--- Superficial injury of wrist and hand (S60) --- Fracture at wrist and hand level (S62)* | *85 (1.7%) 66 (1.3%)* | *140 (1.1%) 99 (0.7%)* | *1.65 ± 1.38 1.5 ± 0.98* | *2.74 ± 1.73 2.52 ± 1.86* | *6 (4.3%) 7 (7.1%)* | *6 (4.3%) 30 (30.3%)* |
| - Injuries to the knee and lower leg (S80-S89) | 123 (2.4%) | 250 (1.9%) | 2.03 ± 1.78 | 3.23 ± 2.26 | 23 (9.2%) | 14 (5.6%) |
| - Injuries to the ankle and foot (S90-S99) | 116 (2.3%) | 235 (1.8%) | 2.03 ± 1.60 | 3.62 ± 3.70 | 16 (6.8%) | 15 (6.4%) |
| - Injuries to unspecified part of trunk, limb or body region (T08-T14) | 275 (5.4%) | 430 (3.2%) | 1.56 ± 1.34 | 2.57 ± 1.84 | 18 (4.2%) | 19 (4.4%) |
| *--- Injury of unspecified body region (T14)* | *252 (5.0%)* | *376 (2.8%)* | *1.49 ± 1.09* | *2.54 ± 1.79* | *18 (4.8%)* | *19 (5.1%)* |
| - Other and unspecified effects of external causes (T66-T78) | 77 (1.5%) | 113 (0.9%) | 1.47 ± 1.12 | 2.50 ± 1.41 | 3 (2.7%) | 3 (2.7%) |
| *--- Adverse effects, not elsewhere classified (T78)* | *65 (1.3%)* | *99 (0.7%)* | *1.52 ± 1.19* | *2.57 ± 1.38* | *3 (3.0%)* | *3 (3.0%)* |
| - Certain early complications of trauma (T79) | 111 (2.2%) | 214 (1.6%) | 1.93 ± 2.28 | 2.80 ± 1.98 | 8 (3.7%) | 3 (1.4%) |

**Table S1d: Number of patients, consultations, comorbid diagnoses, referrals to outpatient care and hospital admissions by ICD10 classification (continued)**

| **ICD10 codes** | **Number of patients** | **Number of consultations** | **Consultations per patient** | **Comorbid diagnoses** | **Outpatient referrals** | **Hospital admissions** |
| --- | --- | --- | --- | --- | --- | --- |
| ***Symptoms, signs and abnormal findings, not elsewhere classified (R00-R99)*** | ***993 (19.6%)*** | ***2,053 (15.4%)*** | ***2.07 ± 2.13*** | ***3.41 ± 2.83*** | ***163 (7.9%)*** | ***155 (7.6%)*** |
| - … involving the digestive system and abdomen (R10-R19) | 351 (6.9%) | 657 (4.9%) | 1.87 ± 1.76 | 3.45 ± 3.57 | 46 (7.0%) | 68 (10.4%) |
| *--- Abdominal and pelvic pain (R10)* | *291 (5.8%)* | *498 (3.7%)* | *1.71 ± 1.36* | *2.54 ± 1.58* | *20 (4.0%)* | *51 (10.2%)* |
| - … involving the circulatory and respiratory systems (R00-R09) | 263 (5.2%) | 492 (3.7%) | 1.87 ± 1.69 | 3.50 ± 2.91 | 41 (8.3%) | 43 (8.7%) |
| *--- Cough (R05) --- Pain in throat and chest (R07)* | *91 (1.8%) 86 (1.7%)* | *146 (1.1%) 132 (1.0%)* | *1.60 ± 1.15 1.53 ± 0.90* | *2.73 ± 1.43 2.55 ± 1.36* | *10 (6.9%) 5 (3.8%)* | *3 (2.1%) 13 (9.9%)* |
| - General symptoms and signs (R50-R69) | 258 (5.1%) | 562 (4.2%) | 2.18 ± 1.95 | 4.20 ± 3.64 | 40 (7.1%) | 36 (6.4%) |
| *--- Headache (R51) --- Oedema, not elsewhere classified (R60)* | *66 (1.3%) 63 (1.3%)* | *131 (1.0%) 145 (1.1%)* | *1.98 ± 1.84 2.30 ± 1.86* | *2.79 ± 1.73 5.08 ± 4.60* | *4 (3.1%) 7 (4.8%)* | *1 (0.8%) 7 (4.8%)* |
| - … involving the skin and subcutaneous tissue (R20-R23) | 107 (2.1%) | 196 (1.5%) | 1.83 ± 1.32 | 3.54 ± 3.45 | 21 (10.7%) | 1 (0.5%) |
| *--- Other skin changes (R23)* | *64 (1.3%)* | *124 (0.9%)* | *1.94 ± 1.49* | *3.15 ± 2.23* | *10 (8.1%)* | *0 (-)* |
| - … involving the nervous and musculoskeletal systems (R25-R29) | 77 (1.5%) | 145 (1.1%) | 1.88 ± 1.70 | 4.23 ± 3.33 | 17 (11.7%) | 4 (2.8%) |
| - … involving cognition, perception, emotional state and behaviour (R40-R46) | 72 (1.4%) | 131 (1.0%) | 1.82 ± 1.83 | 4.03 ± 3.09 | 8 (6.1%) | 6 (4.6%) |
| ***Diseases of the circulatory system (I00-I99)*** | ***923 (18.3%)*** | ***3,202 (24.0%)*** | ***3.47 ± 4.96*** | ***3.55 ± 2.68*** | ***187 (5.8%)*** | ***206 (6.4%)*** |
| - Hypertensive diseases (I10-I15) | 509 (10.1%) | 2,004 (15.0%) | 3.94 ± 5.69 | 3.57 ± 2.70 | 105 (5.2%) | 71 (3.5%) |
| *--- Essential hypertension (I10) --- Hypertensive heart disease (I11)* | *484 (9.6%) 42 (0.8%)* | *1,920 (14.4%) 151 (1.1%)* | *3.97 ± 5.73 3.60 ± 4.61* | *3.58 ± 2.74 4.21 ± 2.66* | *101 (5.3%) 6 (4.0%)* | *64 (3.3%) 10 (6.6%)* |
| - Diseases of veins, lymphatic vessels and lymph nodes, n.e.c. (I80-I89) | 203 (4.0%) | 489 (3.7%) | 2.41 ± 2.59 | 4.01 ± 3.64 | 16 (3.3%) | 53 (10.8%) |
| *--- Varicose veins of lower extremities (I83) --- Phlebitis and thrombophlebitis (I80)* | *103 (2.0%) 62 (1.2%)* | *249 (1.9%) 120 (0.9%)* | *2.42 ± 2.60 1.94 ± 1.48* | *3.58 ± 3.70 4.06 ± 2.72* | *11 (4.4%) 2 (1.7%)* | *9 (3.6%) 20 (16.7%)* |
| - Other forms of heart disease (I30-I52) | 165 (3.3%) | 506 (3.8%) | 3.07 ± 3.12 | 4.65 ± 2.64 | 50 (9.9%) | 48 (9.5%) |

**n.e.c.: not elsewhere classified**

**Table S1e: Number of patients, consultations, comorbid diagnoses, referrals to outpatient care and hospital admissions by ICD10 classification (continued)**

| **ICD10 codes** | **Number of patients** | **Number of consultations** | **Consultations per patient** | **Comorbid diagnoses** | **Outpatient referrals** | **Hospital admissions** |
| --- | --- | --- | --- | --- | --- | --- |
| *--- Heart failure (I50) --- Atrial fibrillation and flutter (I48)* | *75 (1.5%) 47 (0.9%)* | *231 (1.7%) 147 (1.1%)* | *3.08 ± 2.90 3.13 ± 3.19* | *5.23 ± 2.80 5.5 ± 3.43* | *34 (14.7%) 11 (7.5%)* | *26 (11.3%) 24 (16.3%)* |
| - Ischaemic heart diseases (I20-I25) | 155 (3.1%) | 532 (4.0%) | 3.43 ± 4.04 | 4.02 ± 2.72 | 41 (7.7%) | 50 (9.4%) |
| *--- Chronic ischaemic heart disease (I25) --- Acute myocardial infarction (I21)* | *111 (2.2%) 53 (1.1%)* | *353 (2.7%) 202 (1.5%)* | *3.18 ± 3.60 3.81 ± 4.50* | *4.39 ± 2.95 3.72 ± 2.00* | *36 (10.2%) 6 (3.0%)* | *27 (7.7%) 10 (5.0%)* |
| - Cerebrovascular diseases (I60-I69) | 75 (1.5%) | 308 (2.3%) | 4.11 ± 5.36 | 5.25 ± 3.56 | 24 (7.8%) | 18 (5.8%) |
| *--- Stroke, not specified as haemorrhage or infarction (I64)* | *38 (0.8%)* | *159 (1.2%)* | *4.18 ± 5.89* | *4.55 ± 2.18* | *8 (5.0%)* | *9 (5.7%)* |
| - Diseases of arteries, arterioles and capillaries (I70-I79) | 74 (1.5%) | 230 (1.7%) | 3.11 ± 2.46 | 5.53 ± 3.82 | 34 (14.8%) | 24 (10.4%) |
| ***Diseases of the musculoskeletal system and connective tissue (M00-M99)*** | ***870 (17.2%)*** | ***2,056 (15.4%)*** | ***2.36 ± 3.02*** | ***3.29 ± 3.00*** | ***201 (9.8%)*** | ***61 (3.0%)*** |
| - Dorsopathies (M40-M54) | 420 (8.3%) | 904 (6.8%) | 2.15 ± 2.21 | 3.50 ± 3.34 | 73 (8.1%) | 24 (2.7%) |
| --- Other dorsopathies (M50-M54) | 368 (7.3%) | 757 (5.7%) | 2.06 ± 2.01 | 3.28 ± 3.27 | 47 (6.2%) | 14 (1.9%) |
| *----- Dorsalgia (M54)* | *348 (6.9%)* | *703 (5.3%)* | *2.02 ± 1.96* | *3.26 ± 3.32* | *40 (5.7%)* | *10 (1.4%)* |
| --- Spondylopathies (M45-M49) | 51 (1.0%) | 158 (1.2%) | 3.10 ± 3.21 | 6.12 ± 5.18 | 28 (17.7%) | 9 (5.7%) |
| - Arthropathies (M00-M25) | 350 (6.9%) | 857 (6.4%) | 2.45 ± 3.25 | 3.59 ± 3.30 | 103 (12.0%) | 20 (2.3%) |
| --- Other joint disorders (M20-M25) | 221 (4.4%) | 483 (3.6%) | 2.19 ± 2.59 | 3.55 ± 3.52 | 53 (11.0%) | 10 (2.1%) |
| *----- Other joint disorders, not elsewhere classified (M25)* | *165 (3.3%)* | *378 (2.8%)* | *2.29 ± 2.83* | *3.61 ± 3.73* | *31 (8.2%)* | *3 (0.8%)* |
| --- Arthrosis (M15-M19) | 103 (2.0%) | 284 (2.1%) | 2.76 ± 3.75 | 4.00 ± 3.57 | 38 (13.4%) | 9 (3.2%) |
| *----- Gonarthrosis (M17)* | *55 (1.1%)* | *177 (1.3%)* | *3.22 ± 4.75* | *4.14 ± 3.56* | *19 (10.7%)* | *3 (1.7%)* |
| --- Inflammatory polyarthropathies (M05-M14) | 52 (1.0%) | 134 (1.0%) | 2.58 ± 3.20 | 3.66 ± 3.17 | 19 (14.2%) | 3 (2.2%) |
| - Soft tissue disorders (M60-M79) | 216 (4.3%) | 457 (3.4%) | 2.12 ± 1.92 | 4.02 ± 3.78 | 60 (13.1%) | 17 (3.7%) |
| --- Other soft tissue disorders (M70-M79) | 173 (3.4%) | 354 (2.7%) | 2.05 ± 1.89 | 3.37 ± 2.72 | 39 (11.0%) | 6 (1.7%) |

**Table S1f: Number of patients, consultations, comorbid diagnoses, referrals to outpatient care and hospital admissions by ICD10 classification (continued)**

| **ICD10 codes** | **Number of patients** | **Number of consultations** | **Consultations per patient** | **Comorbid diagnoses** | **Outpatient referrals** | **Hospital admissions** |
| --- | --- | --- | --- | --- | --- | --- |
| *----- Other soft tissue disorders, not elsewhere classified (M79)* | *106 (2.1%)* | *218 (1.6%)* | *2.06 ± 1.66* | *3.56 ± 2.95* | *26 (11.9%)* | *3 (1.4%)* |
| ***Diseases of the digestive system (K00-K93)*** | ***742 (14.7%)*** | ***1,672 (12.5%)*** | ***2.25 ± 2.74*** | ***3.67 ± 3.30*** | ***123 (7.4%)*** | ***106 (6.3%)*** |
| - Diseases of oesophagus, stomach and duodenum (K20-K31) | 317 (6.3%) | 689 (5.2%) | 2.17 ± 2.28 | 4.01 ± 3.89 | 47 (6.8%) | 31 (4.5%) |
| *--- Gastro-oesophageal reflux disease (K21) --- Gastritis and duodenitis (K29)* | *173 (3.4%) 126 (2.5%)* | *367 (2.8%) 270 (2.0%)* | *2.12 ± 2.10 2.14 ± 2.24* | *3.66 ± 3.37 4.31 ± 4.56* | *17 (4.6%) 31 (11.5%)* | *8 (2.2%) 13 (4.8%)* |
| - Diseases of oral cavity, salivary glands and jaws (K00-K14) | 199 (3.9%) | 313 (2.4%) | 1.57 ± 1.1± 28 | 2.51 ± 1.87 | 8 (2.6%) | 0 (-) |
| *--- Other disorders of teeth and supporting structures (K08)* | *166 (3.3%)* | *260 (2.0%)* | *1.57 ± 1.16* | *2.38 ± 1.39* | *6 (2.3%)* | *0 (-)* |
| - Other diseases of intestines (K55-K64) | 128 (2.5%) | 273 (2.1%) | 2.13 ± 1.83 | 4.71 ± 4.57 | 37 (13.6%) | 25 (9.2%) |
| *--- Haemorrhoids and perianal venous thrombosis (K64)* | *66 (1.3%)* | *149 (1.1%)* | *2.26 ± 1.96* | *4.66 ± 5.07* | *19 (12.8%)* | *15 (10.1%)* |
| - Diseases of liver (K70-K77) | 92 (1.8%) | 275 (2.1%) | 2.99 ± 3.56 | 5.51 ± 4.15 | 29 (10.6%) | 35 (12.7%) |
| *--- Alcoholic liver disease (K70)* | *67 (1.3%)* | *197 (1.5%)* | *2.94 ± 3.76* | *5.60 ± 4.36* | *24 (12.2%)* | *33 (16.8%)* |
| - Hernia (K40-K46) | 58 (1.2%) | 111 (0.8%) | 1.91 ± 1.85 | 4.93 ± 6.17 | 13 (11.7%) | 16 (14.4%) |
| - Disorders of gallbladder, biliary tract and pancreas (K80-K87) | 51 (1.0%) | 201 (1.5%) | 3.94 ± 4.32 | 5.38 ± 4.48 | 21 (10.5%) | 18 (9.0%) |
| ***Diseases of the skin and subcutaneous tissue (L00-L99)*** | ***677 (13.4%)*** | ***1,443 (10.8%)*** | ***2.13 ± 2.36*** | ***3.12 ± 2.80*** | ***94 (6.5%)*** | ***55 (3.8%)*** |
| - Infections of the skin and subcutaneous tissue (L00-L08) | 221 (4.4%) | 385 (2.9%) | 1.74 ± 1.31 | 2.95 ± 3.01 | 17 (4.4%) | 25 (6.5%) |
| *--- Cellulitis (L03) --- Impetigo (L01)* | *92 (1.8%) 76 (1.5%)* | *175 (1.3%) 124 (0.9%)* | *1.90 ± 1.38 1.63 ± 1.33* | *3.49 ± 4.09 2.56 ± 1.53* | *16 (9.1%) 0 (-)* | *19 (10.9%) 5 (4.0%)* |
| - Dermatitis and eczema (L20-L30) | 208 (4.1%) | 407 (3.1%) | 1.96 ± 1.84 | 3.72 ± 3.45 | 31 (7.6%) | 9 (2.2%) |
| *--- Pruritus (L29)* | *99 (2.0%)* | *180 (1.4%)* | *1.82 ± 1.60* | *3.66 ± 3.68* | *5 (2.8%)* | *4 (2.2%)* |
| - Other disorders of the skin and subcutaneous tissue (L80-L99) | 154 (3.1%) | 339 (2.5%) | 2.20 ± 2.34 | 3.09 ± 2.20 | 11 (3.2%) | 9 (2.7%) |
| *--- Other disorders of skin and subcutaneous tissue, n.e.c. (L98)* | *63 (1.3%)* | *105 (0.8%)* | *1.67 ± 1.12* | *2.99 ± 2.68* | *1 (1.0%)* | *5 (4.8%)* |

**n.e.c.: not elsewhere classified**

**Table S1g: Number of patients, consultations, comorbid diagnoses, referrals to outpatient care and hospital admissions by ICD10 classification (continued)**

| **ICD10 codes** | **Number of patients** | **Number of consultations** | **Consultations per patient** | **Comorbid diagnoses** | **Outpatient referrals** | **Hospital admissions** |
| --- | --- | --- | --- | --- | --- | --- |
| - Papulosquamous disorders (L40-L45) | 80 (1.6%) | 245 (1.8%) | 3.06 ± 3.71 | 2.90 ± 2.09 | 20 (8.2%) | 11 (4.5%) |
| *--- Psoriasis (L40)* | *79 (1.6%)* | *243 (1.8%)* | *3.08 ± 3.73* | *2.91 ± 2.10* | *20 (8.2%)* | 11 (4.5%) |
| - Disorders of skin appendages (L60-L75) | 58 (1.2%) | 99 (0.7%) | 1.71 ± 1.49 | 4.62 ± 6.09 | 15 (15.2%) | 2 (2.0%) |
| ***Endocrine, nutritional and metabolic diseases (E00-E90)*** | ***456 (9.0%)*** | ***1,755 (13.2%)*** | ***3.85 ± 5.67*** | ***3.82 ± 3.11*** | ***109 (6.2%)*** | ***64 (3.7%)*** |
| - Diabetes mellitus (E10-E14) | 298 (5.9%) | 1,234 (9.3%) | 4.14 ± 6.39 | 3.29 ± 2.17 | 48 (3.9%) | 64 (3.7%) |
| *--- Non-insulin-dependent diabetes mellitus (E11) --- Insulin-dependent diabetes mellitus (E10)* | *256 (5.1%) 46 (0.9%)* | *1,032 (7.7%) 197 (1.5%)* | *4.03 ± 6.10 4.28 ± 6.75* | *3.39 ± 2.23 2.76 ± 1.73* | *42 (4.1%) 4 (2.0%)* | *47 (3.8%) 43 (4.2%)* |
| - Metabolic disorders (E70-E90) | 114 (2.3%) | 425 (3.2%) | 3.73 ± 4.66 | 4.80 ± 3.57 | 37 (8.7%) | 4 (2.0%) |
| *--- Disorders of lipoprotein metabolism and other lipidaemias (E78)* | *77 (1.5%)* | *332 (2.5%)* | *4.31 ± 5.37* | *4.28 ± 2.45* | *24 (7.2%)* | *10 (2.4%)* |
| - Disorders of thyroid gland (E00-E07) | 50 (1.0%) | 167 (1.3%) | 3.34 ± 2.80 | 4.82 ± 4.59 | 22 (13.2%) | 7 (2.1%) |
| ***Diseases of the nervous system (G00-G99)*** | ***394 (7.8%)*** | ***994 (7.5%)*** | ***2.52 ± 3.00*** | ***4.16 ± 3.60*** | ***67 (6.7%)*** | ***56 (5.6%)*** |
| - Episodic and paroxysmal disorders (G40-G47) | 299 (5.9%) | 722 (5.4%) | 2.41 ± 2.37 | 4.11 ± 3.87 | 46 (6.4%) | 37 (5.1%) |
| *--- Sleep disorders (G47) --- Epilepsy (G40) --- Other headache syndromes (G44)* | *134 (2.7%) 101 (2.0%) 52 (1.0%)* | *308 (2.3%) 275 (2.1%) 108 (0.8%)* | *2.30 ± 1.95 2.72 ± 2.75 2.08 ± 2.29* | *4.63 ± 4.33 3.68 ± 3.06 5.07 ± 5.80* | *23 (7.5%) 19 (6.9%) 4 (3.7%)* | *13 (4.2%) 17 (6.2%) 3 (2.8%)* |
| ***Diseases of the genitourinary system (N00-N99)*** | ***213 (4.2%)*** | ***464 (3.5%)*** | ***2.18 ± 2.34*** | ***3.78 ± 3.31*** | ***48 (10.3%)*** | ***35 (7.5%)*** |
| - Other diseases of urinary system (N30-N39) | 95 (1.9%) | 162 (1.2%) | 1.71 ± 1.50 | 2.79 ± 2.09 | 18 (11.1%) | 6 (3.7%) |
| *--- Other disorders of urinary system (N39)* | *80 (1.6%)* | *132 (1.0%)* | *1.65 ± 1.43* | *2.80 ± 2.10* | *14 (10.6%)* | *4 (3.0%)* |
| - Diseases of male genital organs (N40-N51) | 64 (1.3%) | 145 (1.1%) | 2.27 ± 2.35 | 4.44 ± 3.74 | 16 (11.0%) | 13 (9.0%) |
| ***Factors influencing health status and contact with health services (Z00-Z99)*** | ***184 (3.6%)*** | ***350 (2.6%)*** | ***1.90 ± 1.78*** | ***4.13 ± 3.49*** | ***15 (4.3%)*** | ***20 (5.7%)*** |
| - Potential health hazards related to communicable diseases (Z20-Z29) | 69 (1.4%) | 143 (1.1%) | 2.07 ± 2.07 | 4.48 ± 3.94 | 5 (3.5%) | 4 (2.8%) |

**Table S1h: Number of patients, consultations, comorbid diagnoses, referrals to outpatient care and hospital admissions by ICD10 classification (continued)**

| **ICD10 codes** | **Number of patients** | **Number of consultations** | **Consultations per patient** | **Comorbid diagnoses** | **Outpatient referrals** | **Hospital admissions** |
| --- | --- | --- | --- | --- | --- | --- |
| ***Diseases of the eye and adnexa (H00-H59)*** | ***169 (3.3%)*** | ***317 (2.4%)*** | ***1.88 ± 1.68*** | ***2.83 ± 2.03*** | ***22 (6.9%)*** | ***9 (2.8%)*** |
| - Other disorders of eye and adnexa (H55-H59) | 58 (1.2%) | 90 (0.7%) | 1.55± 1.06 | 2.3 ± 1.18 | 6 (6.7%) | 3 (3.3%) |
| --- Other disorders of eye and adnexa (H57) | 58 (1.2%) | 90 (0.7%) | 1.55 ± 1.06 | 2.3 ± 1.18 | 6 (6.7%) | 3 (3.3%) |
| ***Diseases of the ear and mastoid process (H60-H95)*** | ***150 (3.0%)*** | ***252 (1.9%)*** | ***1.68 ± 1.56*** | ***3.29 ± 3.01*** | ***32 (12.7%)*** | ***3 (1.2%)*** |
| - Diseases of external ear (H60-H62) | 66 (1.3%) | 102 (0.8%) | 1.55 ± 1.06 | 2.57 ± 1.75 | 6 (5.9%) | 1 (1.0%) |
| --- Other disorders of external ear (H61) | 52 (1.0%) | 74 (0.6%) | 1.42 *±* 0.91 | 2.51 *±* 1.92 | 4 (5.4%) | 0 (-) |
| - Other disorders of ear (H90-H95) | 52 (1.0%) | 98 (0.7%) | 1.88 ± 2.18 | 4.54 ± 3.99 | 18 (18.4%) | 1 (1.0%) |
| ***Neoplasms (C00-D48)*** | ***101 (2.0%)*** | ***226 (1.7%)*** | ***2.24 ± 2.27*** | ***4.55 ± 3.68*** | ***29 (12.8%)*** | ***23 (10.2%)*** |
| - Malignant neoplasms (C00-C97) | 68 (1.4%) | 169 (1.3%) | 2.49 ± 2.58 | 4.86 ± 4.07 | 24 (14.2%) | 16 (9.5%) |
| ***Diseases of blood, blood-forming organs and immune mechanism (D50-D89)*** | ***66 (1.3%)*** | ***172 (1.3%)*** | ***2.61 ± 1.89*** | ***5.24 ± 4.22*** | ***21 (12.2%)*** | ***10 (5.8%)*** |
